# GSK3β Serine 389 Phosphorylation Modulates Cardiomyocyte Hypertrophy and Ischemic Injury

**DOI:** 10.3390/ijms222413586

**Published:** 2021-12-18

**Authors:** Laura Vainio, Saija Taponen, Sini M. Kinnunen, Eveliina Halmetoja, Zoltan Szabo, Tarja Alakoski, Johanna Ulvila, Juhani Junttila, Päivi Lakkisto, Johanna Magga, Risto Kerkelä

**Affiliations:** 1 Research Unit of Biomedicine, Department of Pharmacology and Toxicology, University of Oulu, Oulu 90220, Finland; laura.vainio@oulu.fi (L.V.); saija.taponen@gmail.com (S.T.); sini.m.kinnunen@helsinki.fi (S.M.K.); eveliina.halmetoja@oulu.fi (E.H.); zoltan.szabo@oulu.fi (Z.S.); tarja.alakoski@oulu.fi (T.A.); joulvila@gmail.com (J.U.); Johanna.Magga@oulu.fi (J.M.); 2 Biocenter Oulu, University of Oulu, Oulu 90220, Finland; juhani.junttila@oulu.fi; 3 Drug Research Program, Division of Pharmacology and Pharmacotherapy, Faculty of Pharmacy, University of Helsinki, Helsinki 00014, Finland; 4 Medical Research Center Oulu, Oulu University Hospital and University of Oulu, Oulu 90220, Finland; 5 Research Unit of Internal Medicine, Division of Cardiology, Oulu University Hospital and University of Oulu, Oulu 90220, Finland; 6 Unit of Cardiovascular Research, Minerva Institute for Medical Research, Helsinki 00014, Finland; paivi.lakkisto@helsinki.fi; 7 Department of Clinical Chemistry and Hematology, University of Helsinki and Helsinki University Hospital, Helsinki 00014, Finland

**Keywords:** glycogen synthase kinase 3β, cardiomyocyte hypoxia, cardiomyocyte hypertrophy, cell death

## Abstract

Prior studies show that glycogen synthase kinase 3β (GSK3β) contributes to cardiac ischemic injury and cardiac hypertrophy. GSK3β is constitutionally active and phosphorylation of GSK3β at serine 9 (S9) inactivates the kinase and promotes cellular growth. GSK3β is also phosphorylated at serine 389 (S389), but the significance of this phosphorylation in the heart is not known. We analyzed GSK3β S389 phosphorylation in diseased hearts and utilized overexpression of GSK3β carrying ser→ala mutations at S9 (S9A) and S389 (S389A) to study the biological function of constitutively active GSK3β in primary cardiomyocytes. We found that phosphorylation of GSK3β at S389 was increased in left ventricular samples from patients with dilated cardiomyopathy and ischemic cardiomyopathy, and in hearts of mice subjected to thoracic aortic constriction. Overexpression of either GSK3β S9A or S389A reduced the viability of cardiomyocytes subjected to hypoxia–reoxygenation. Overexpression of double GSK3β mutant (S9A/S389A) further reduced cardiomyocyte viability. Determination of protein synthesis showed that overexpression of GSK3β S389A or GSK3β S9A/S389A increased both basal and agonist-induced cardiomyocyte growth. Mechanistically, GSK3β S389A mutation was associated with activation of mTOR complex 1 signaling. In conclusion, our data suggest that phosphorylation of GSK3β at S389 enhances cardiomyocyte survival and protects from cardiomyocyte hypertrophy.

## 1. Introduction

Cardiovascular diseases are the leading cause of morbidity and mortality in the world [1,2]. New therapeutic approaches are critically needed. Glycogen synthase kinase-3β (GSK3β), which is a constitutively active serine/threonine protein kinase found in 1980 [3] is involved in regulation of multiple cellular functions such as embryonic development, energy metabolism, cell motility, apoptosis, and cell differentiation and proliferation [4,5]. In the heart, GSK3 has been shown to play an important role in cardiac ischemic injury and cardiac hypertrophy [6,7].

GSK3β is active in unstimulated cells, where it phosphorylates and thus inhibits growth stimulating substrates. There are more than 40 substrates for GSK3 and an additional 500 other potential candidates have been identified [8,9]. GSK3 is regulated through phosphorylation, through complex formation and intracellular localization [10]. Phosphorylation of GSK3β at serine 9 (S9) inactivates the kinase and has been shown to regulate hypertrophic cardiomyocyte growth and viability [7]. However, the biological function of other GSK3β phosphorylation sites in cardiomyocytes is not well understood. In 2008, Thornton et al. reported a complementary phosphorylation site of GSK3β, serine 389 (S389), which also inactivates the kinase by direct phosphorylation [11]. Subsequent studies showed that this phosphorylation site participates in the regulation of lymphocyte and neuron survival [12,13].

The key upstream regulator of GSK3β is PI3K-Akt pathway, which is activated by multiple growth factors that couple their cell surface receptors to specific PI3K isoforms. Activated Akt phosphorylates GSK3β at S9 residue, which inactivates the kinase and allows cardiomyocyte hypertrophic growth to occur [6]. In addition to Akt, mTOR (mammalian/mechanistic target of rapamycin)-p70S6K (p70 ribosomal protein S6 kinase) pathway also leads to phosphorylation and inactivation of GSK3 [14,15]. Reciprocally, GSK3β can activate mTOR through TSC1/2 [16]. The mTOR pathway is an important regulator of both physiological and pathological processes in the heart [16] and is found in two distinct complexes, mTORC1 and mTORC2. Manipulation of these complexes can conduct both protective and deleterious effects in cardiac remodeling, depending on the model [17,18,19,20,21]. Additionally, mTOR contributes to cardiomyocyte survival; inhibition of mTORC1 showed protection against cardiac ischemia/reperfusion injury, while dual mTOR inhibitors abolished cardioprotection after ischemic preconditioning [16,22].

The aim of this study was to investigate the significance of phosphorylation of GSK3β at S389 in the myocardium. We found that phosphorylation of GSK3β at S389 was increased in diseased human and mouse hearts. Studies in primary rat cardiomyocytes showed that GSK3β S389 phosphorylation enhances cardiomyocyte survival and protects from cardiomyocyte hypertrophy. Analysis for potential downstream mechanisms indicates that GSK3β S389 regulates the activation of mTORC1 signaling.

## 2. Results

### 2.1. Phosphorylation of GSK3β S389 Is Increased in Diseased Human and Rodent Hearts

**Figure 1:**
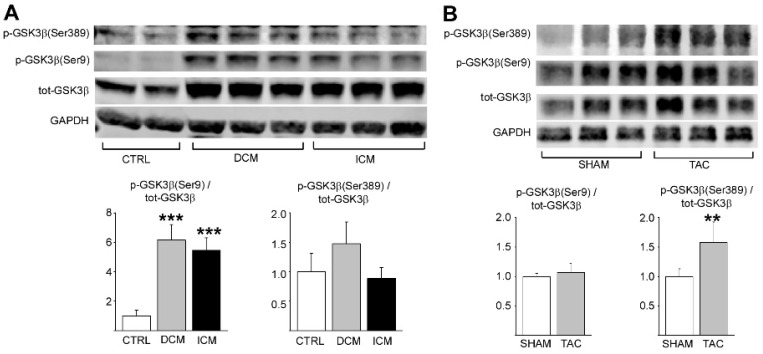
Analysis for phosphorylation of GSK3β in diseased human and mouse hearts. (**A**) Western blot analysis of phosphorylated glycogen synthase kinase 3β (GSK3β) at Ser9 and Ser389 and total GSK3β in left ventricular samples from human dilated cardiomyopathy (DCM) and ischemic cardiomyopathy (ICM) hearts. *** *p* < 0.001 compared to control. (**B**) Western blot analysis of phosphorylated GSK3β and total GSKβ in left ventricular samples of mice subjected to transverse aortic constriction (TAC) for 6 weeks. ** *p* < 0.01 compared to sham. Glyceraldehyde 3-phosphate dehydrogenase (GAPDH) was used as loading control; *n* = 4–6 for each group.

We first investigated if phosphorylation of GSK3β is dysregulated in left ventricular samples of explanted hearts from patients with end-stage dilated or ischemic cardiomyopathy. In comparison, GSK3β phosphorylation was analyzed from left ventricular samples of accidental death victims with no history of cardiovascular disease or evidence of cardiovascular disease at autopsy. We found that phosphorylation of GSK3β at S389 was increased both in dilated and ischemic cardiomyopathy hearts (Figure 1A). In addition, S9 phosphorylated form of GKS3β was increased in cardiomyopathy hearts (Figure 1A).

We then analyzed if phosphorylation of GSK3β is altered in experimental heart failure model in mice. Wild type mice were subjected to TAC for 6 weeks and left ventricular tissue samples were collected for analysis. Western blot analysis showed that phosphorylation of S389 in GSK3β was increased in response to chronic hemodynamic pressure overload by TAC (Figure 1B). On the other hand, cardiac stress had no effect on phosphorylation of S9 residue (Figure 1B). The difference in GSK3β phosphorylation status between the diseased human and mouse myocardium may stem from the difference in the type of stress (i.e., volume overload vs. pressure overload) or from the duration of the stress (years vs. weeks).

### 2.2. GSK3β S389 Phosphorylation Promotes GSK3β S9 Phosphorylation

As phosphorylation of GSK3β at S389 was augmented in diseased hearts in both human and mice, we wanted to examine the biological role of GSK3β phosphorylation in cardiomyocytes more closely. For this, we utilized adenoviral overexpression of GSK3β with mutations on key phosphorylation sites of the kinase. Neonatal rat ventricular cardiomyocytes were transduced with adenoviruses encoding for wildtype GSK3β (WT), GSK3β with serine 9 to alanine mutation (S9A), GSK3β with serine 389 to alanine mutation (S389A), and double mutated GSK3β with S9A and S389A mutations (S9A/S389A). LacZ was used as a control virus.

**Figure 2:**
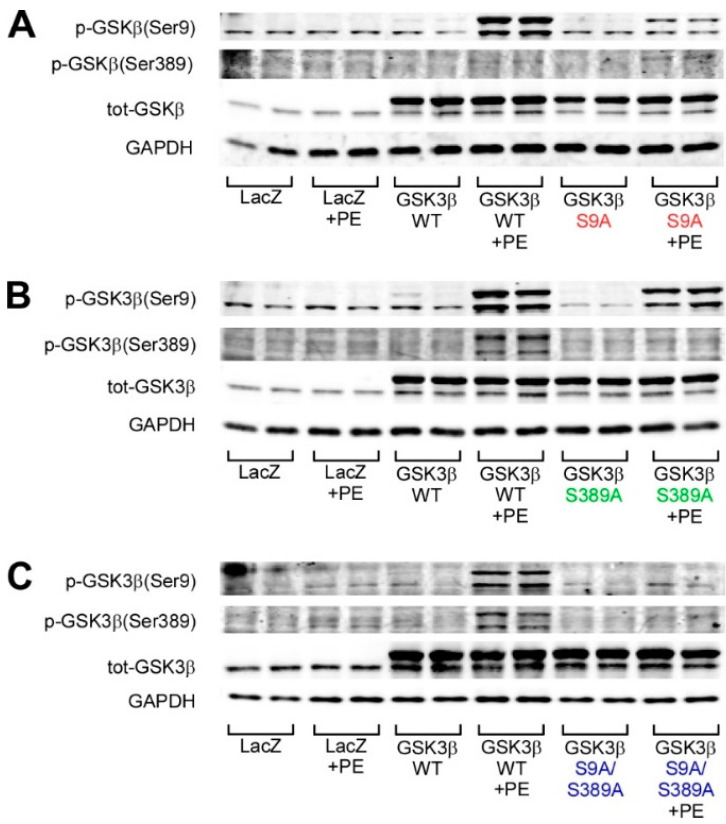
Validation of adenoviruses encoding for GSK3β S9A, GSK3β S389A, and GSK3β S9A/S389A. Neonatal rat cardiomyocytes were infected with LacZ, wild-type (WT) GSK3β, or GSK3β carrying Ser→Ala mutation at Ser9 and Ser389. Phenylephrine (PE) was used to stimulate GSK3β phosphorylation where indicated. Shown is Western blot analysis of phosphorylated GSK3β at Ser9, Ser389, and for total GSK3β. (**A**) Overexpression of GSK3β S9A and PE stimulus. (**B**) Overexpression of GSK3β S389A and PE stimulus. (**C**) Overexpression of GSK3β S9A/S389A and PE stimulus. Glyceraldehyde 3-phosphate dehydrogenase (GAPDH) was used as loading control; *n* = 4 for each group.

We first assessed for the possible regulation of GSK3β S9 phosphorylation by GSK3β S389. Analysis for GSK3β phosphorylation shows that phenylephrine (PE) only induces weak phosphorylation of GSK3β at S9 and S389 in cardiomyocytes transduced with LacZ (Figure 2A–C). Cells overexpressing WT GSK3β show increased phosphorylation of GSK3β at both S9 and S389 in response to PE. As expected, overexpression of GSK3β S9A attenuates PE-induced GSK3β S9 phosphorylation when compared to cells overexpressing WT GSK3β (Figure 2A). In Figure 2A, enhanced GSK3β Ser9 phosphorylation should not be detected in GSK3β S9A overexpressing cells as the mutated phosphorylation site cannot be phosphorylated. The noted signal for GSK3β S9 phosphorylation in GSK3β S9A overexpressing cells (Figure 2A, lanes 11–12) possibly represents nonspecific binding of the antibody in samples with high levels of GSK3β.

Cells overexpressing GSK3β S389A show abolished basal and PE-induced GSK3β S389 phosphorylation and, interestingly, also reduction in the basal and PE-induced GSK3β S9 phosphorylation (Figure 2B). On the other hand, mutation of GSK3β at S9A does not reduce the phosphorylation of GSK3β at S389 (Figure 2A). Overexpression of the double mutant GSK3β (S9A/S389A) attenuates both the basal and PE-induced phosphorylation of the phosphorylation sites (Figure 2C). The phosphorylation of GSK3β at S389 thus augments the phosphorylation of GSK3β at S9.

### 2.3. GSK3β S389 Regulates Cardiomyocyte Viability

**Figure 3:**
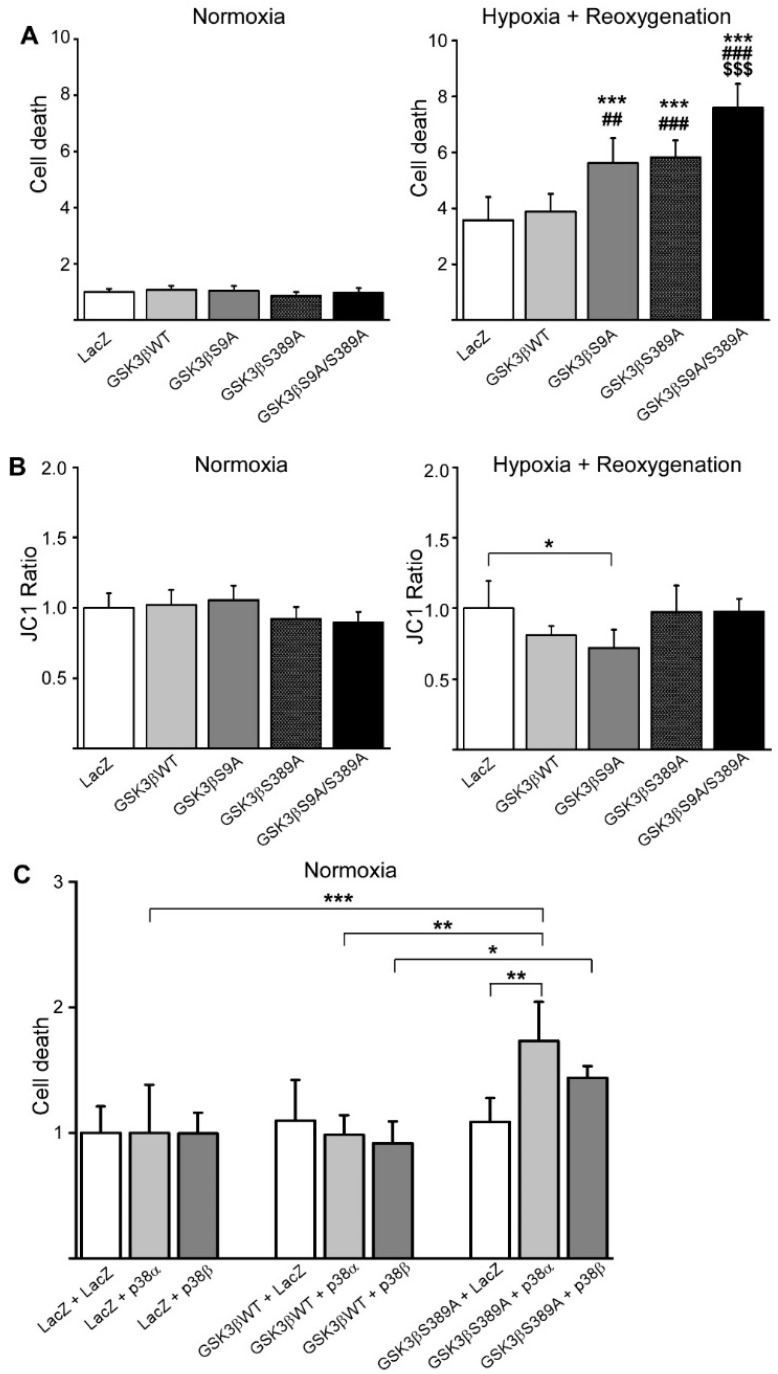
Overexpression of GSK3β S389A reduces cardiomyocyte viability. Cultured adult rat cardiomyocytes were infected with adenoviruses indicated in the figure. (**A**) Twenty-four hours later, cells were subjected to hypoxia–reoxygenation and cell viability was measured with adenylate kinase release. Cells cultivated in normoxia were used as a control. *** *p* < 0.001 compared to control LacZ, ^##^ *p* < 0.01, ^###^ *p* < 0.001 compared to GSK3β WT, ^$$$^ *p* < 0.001 compared to GSK3β S9A and S389A. (**B**) Analysis for mitochondrial membrane potential by JC1 assay in normoxia and following hypoxia–reoxygenation. * *p* < 0.05. (**C**) Cardiomyocytes were infected with adenoviruses encoding for LacZ, p38α, p38β GSK3β WT, and S389A as indicated, and cell viability was measured with adenylate kinase release. * *p* < 0.05, ** *p* < 0.01, *** *p* < 0.001; *n* = 5–6 for each group.

To study the significance of GSK3β S389 phosphorylation in regulating cardiomyocyte viability, we transduced adult rat cardiomyocytes with WT GSK3β or GSK3β variants and analyzed cell death by measuring the release of adenylate kinase from necrotic cardiomyocytes [23]. Overexpression of WT GSK3β or GSK3β variants had no effect on cardiomyocyte viability in normoxic conditions (Figure 3A). As ischemic conditions are major reasons for cardiomyocyte death, we subjected the cardiomyocytes to hypoxia–reoxygenation injury. Analysis of cell death showed that in cardiomyocytes subjected to hypoxia–reoxygenation, cell viability was decreased by overexpression of either GSK3β S9A or GSK3β S389A when compared with LacZ or WT GSK3β infected cardiomyocytes (Figure 3A). In cardiomyocytes overexpressing GSK3β S9A/S389A, cell viability was further reduced when compared to cells overexpressing GSK3β S9A or GSK3β S389A (Figure 3A).

JC1 assay was performed to investigate the effect of GSK3β on mitochondrial membrane potential. Overexpression of WT GSK3β or GSK3β variants had no effect on mitochondrial membrane potential in cardiomyocytes at normoxia (Figure 3B). In cardiomyocytes subjected to hypoxia–reoxygenation, overexpression of GSK3β S9A significantly enhanced the mitochondrial membrane potential transition, increasing the probability of mitochondrial pore opening (Figure 3B). Overexpression of GSK3β S389A or GSK3β S9A/S389A had no effect on mitochondrial membrane potential compared to LacZ transduced cardiomyocytes. Phosphorylation of GSK3β at S389 thus enhances cardiomyocyte survival upon hypoxia–reoxygenation, but that does not involve regulation of mitochondrial membrane potential.

p38 MAPK is activated in response to cardiac ischemia and previous data suggest that inhibition of p38 during ischemia/reperfusion has a cardioprotective effect [24,25,26]. To address if GSK3β S389A mediates the cardiomyocyte survival downstream of p38 pathway, we transduced cardiomyocytes with adenoviruses encoding for p38α or p38β together with WT GSK3β or GSK3β S389A. Analysis for adenylate kinase release showed that p38α or p38β had no effect on the cardiomyocyte survival in cells overexpressing LacZ or WT GSK3β (Figure 3C). On the other hand, overexpression of p38α significantly induced cell death in cardiomyocytes overexpressing GSK3β S389A (Figure 3C).

### 2.4. GSK3β S389 Regulates Cardiomyocyte Growth

**Figure 4:**
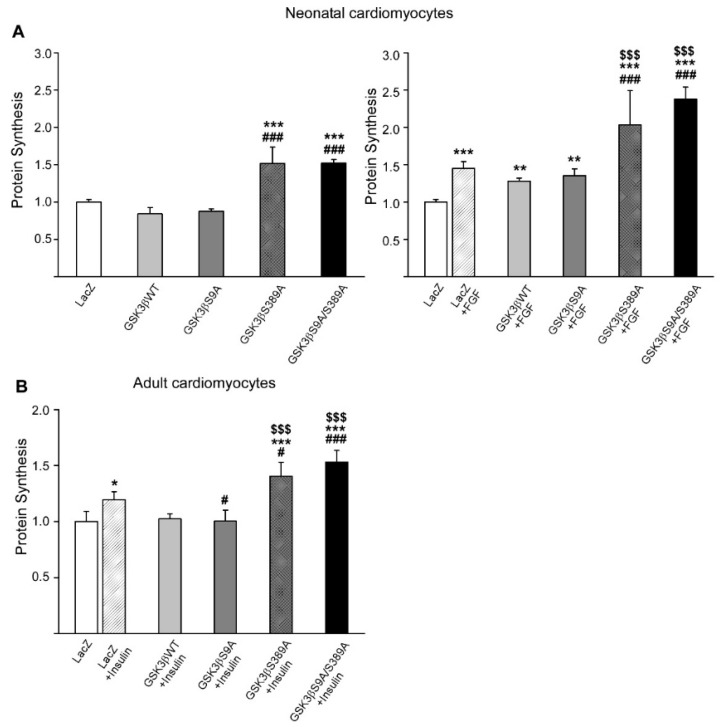
Overexpression of GSK3β S389A enhances protein synthesis in cardiomyocytes. Cultured rat cardiomyocytes were infected with adenoviruses as depicted in the figure, and 24 h later treated with FGF (20 ng/mL) or insulin (20 µg/mL) for 24 h. (**A**) Shown is analysis for [^3^H]-leucine incorporation in neonatal cardiomyocytes. Left panel, *** *p* < 0.001 compared to LacZ, ^###^ *p* < 0.001 compared to GSKβ WT and GSK3β S9A. Right panel, ** *p* < 0.01, *** *p* < 0.001 compared to LacZ, ^###^ *p* < 0.001 compared to LacZ + FGF, ^$$$^ *p* < 0.001 compared to GSK3β WT + FGF, GSK3β S9A + FGF. (**B**) Shown is analysis for [^3^H]-leucine incorporation in adult cardiomyocytes. * *p* < 0.05, *** *p* < 0.001 compared to LacZ, ^#^ *p* < 0.05, ^###^ *p* < 0.001 compared to LacZ + insulin, ^$$$^ *p* < 0.001 compared to GSK3β WT + insulin and GSK3β S9A + insulin; *n* = 4 for each group.

To investigate the role of GSK3β S389 in regulating hypertrophic cardiomyocyte growth, we overexpressed GSK3β variants in adult and neonatal cardiomyocytes and induced hypertrophy with insulin or FGF. Analysis for cardiomyocyte hypertrophy by measuring for [^3^H]-leucine incorporation showed that overexpression of GSK3β S389A or GSK3β S9A/S389A induced both basal and FGF-induced protein synthesis in neonatal cardiomyocytes (Figure 4A). On the other hand, overexpression of WT GSK3β or GSK3β S9A had no effect on the basal or FGF-induced protein synthesis (Figure 4A). Consistent with the data in neonatal cardiomyocytes, overexpression of GSK3β S389A and GSK3β S9A/S389A in adult cardiomyocytes significantly augmented the agonist-induced protein synthesis (Figure 4B). Overexpression of GSK3β S9A, on the other hand, decreased insulin-induced protein synthesis compared to the LacZ transduced cardiomyocytes (Figure 4B).

### 2.5. GSK3β S389 Regulates mTORC1 Activity

**Figure 5:**
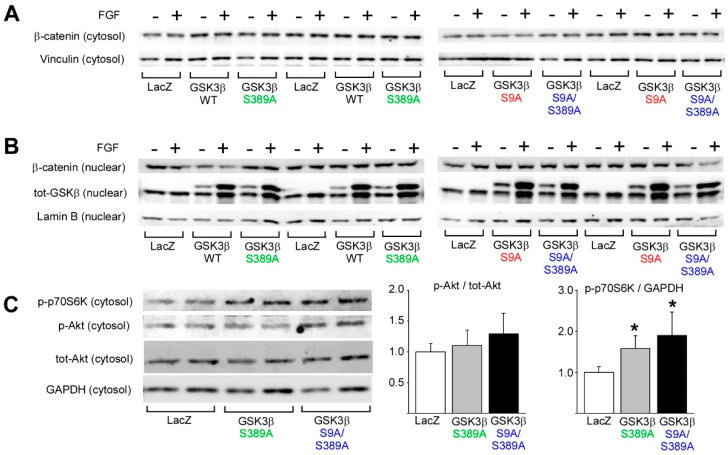
GSK3β S389 regulates p70S6 kinase. Cultured neonatal rat cardiomyocytes were infected with adenoviruses depicted in the figure, and 24 h later treated with FGF (20 ng/mL) for 24 h where indicated. (**A**) Western blot analysis of β-catenin in cytosolic fractions. Vinculin was used as loading control. (**B**) Western blot analysis for β-catenin and total GSK3β in nuclear fractions. Lamin B was used as a loading control. (**C**) Western blot analysis of phosphorylated p70S6 kinase (Thr389) and phosphorylated Akt (Ser473) in cytosol. * *p* < 0.05 compared to LacZ. Glyceraldehyde 3-phosphate dehydrogenase (GAPDH) was used as a loading control; *n* = 4 for each group.

β-catenin is a central downstream effector of GSK3β. Cytosolic β-catenin levels are regulated by a multimolecular complex that also includes GSK3β. Phosphorylation of β-catenin by GSK3β targets β-catenin for ubiquitination and degradation by the proteasomes [27]. Western blot analysis of β-catenin levels in cardiomyocytes showed that overexpression of WT GSK3β or GSK3β harboring S9A/S389A mutations had no effect on the cytocolic levels of β-catenin (Figure 5A). In addition, stimulation of cardiomyocytes with FGF did not affect the β-catenin levels in the cytosol. These data thus suggest that β-catenin does not mediate the effects of GSK3β S389 phosphorylation in isolated cardiomyocytes.

Consistent with the lack of effect of GSK3β on cytosolic levels of β-catenin, overexpression of GSK3β or GSK3β harboring S9A/S389A mutations had no effect on the levels of nuclear β-catenin (Figure 5B). Analysis of nuclear translocation of GSK3β showed that stimulation of cardiomyocytes by FGF induced robust translocation of GSK3β to the nucleus. Overexpression of GSK3β S389A had no discernible effect on the GSK3β localization to the nucleus when compared to WT GSK3β (Figure 5B, left panels), whereas mutation of GSK3β at both S9 and S389 resulted in a modest reduction in nuclear localization of GSK3β at basal state when compared to GSK3β S9A (Figure 5B, right panels).

PI3K-Akt-mTOR signaling network is the central regulatory mechanism controlling cell growth. While Akt is the major regulator of GSK3 activity, prior studies indicate that GSK3 can regulate mTOR activity in some circumstances [28,29]. One of the central effectors of growth promoting mTOR Complex 1 (mTORC1) is p70 ribosomal protein S6 kinase (p70S6K). To investigate if the increased cardiomyocyte hypertrophy in GSK3β S389A overexpressing cardiomyocytes is associated with activation of mTORC1, we analyzed for phosphorylation of p70S6K. Western blot analysis showed that phosphorylation of p70S6K was increased by overexpression of both GSK3β S389A and GSK3β S9A/S389A (Figure 5C). Interestingly, overexpression of GSK3β S9A/S389A also showed some evidence of increased Akt phosphorylation, but that did not reach significance (Figure 5C). The increase in p70S6K phosphorylation thus suggests that phosphorylation of GSK3β at S389 regulates mTORC1 activity.

## 3. Discussion

### 3.1. GSK3β S389 Phosphorylation Is Cardioprotective

Prior studies have shown that inhibition of GSK3β through the phosphorylation of S9 is important for cardioprotection after ischemia–reperfusion [30,31,32]. In a recent study, Thornton et al. showed with GSK3β Ser389Ala mutant mice, that failure to inactivate nuclear GSK3β by Ser389 phosphorylation causes neuronal cell death in subregions of the hippocampus and cortex [13]. By using the same mouse line, they also demonstrated that phosphorylation of GSK3β at S389 is important for the survival of lymphocytes during DNA double-strand break response [12].

To study the contribution of GSK3β S389 in regulating cardiomyocyte viability, we utilized overexpression of different GSK3β-variants in adult rat cardiomyocytes and subjected the cells to hypoxia–reoxygenation injury. We found that overexpression of GSK3β S9A and S389A reduced viability of cardiomyocytes subjected to hypoxia–reoxygenation injury and overexpression of S9A/S389A further augmented cell death, indicating that the S389 phosphorylation site regulates hypoxia–reoxygenation response in cardiomyocytes. It has been suggested that delaying the opening of mitochondrial permeability transition pore (mPTP) is the mechanism of cardioprotection afforded by GSK3β inhibition [30]. Data from cardiac specific transgenic mouse model showed that if S9 is mutated to alanine and GSK3β cannot be inhibited, postconditioning cannot salvage myocardium from ischemia–reperfusion as in wild-type mice, because constitutively active GSK3β is unable to inhibit the opening of mPTP [33]. On the other hand, in the GSK3α/β knock-in mice of Nishino et al., in which the PKB/Akt phosphorylation sites on GSK3α (Ser 21) and GSK3β (Ser9) were mutated to alanine, pre- and postconditioning did protect mice from ischemia–reperfusion [34]. In the current study, overexpression of GSK3β S9A reduced the viability of cardiomyocytes subjected to hypoxia–reoxygenation injury, and the analysis for mitochondrial membrane potential showed increased probability of mPTP opening in cells overexpressing GSK3β S9A. However, the increased cell death in cardiomyocytes overexpressing GSK3β S389A or GSK3β S9A/S389A was not associated with a decrease in mitochondrial membrane potential. This suggests that the cellular protection conferred by phosphorylation of GSK3β S389 is mediated by other mechanisms.

GSK3β phosphorylation site at S389 is phosphorylated by p38 MAPK and this also inactivates GSK3β [11]. Data by Zhang and coworkers also suggest that adiponectin induces GSK3β Ser389 phosphorylation via stimulation of the p38 MAPK signaling pathway [35]. We found that overexpression of p38α augmented cell death in cardiomyocytes transduced with GSK3β S389A, but not in the cells transduced with LacZ or WT GSK3β. These data suggest that phosphorylation of GSK3β at Ser389 is necessary in protecting from cell death in response to p38 pathway activation.

### 3.2. GSK3β S389 Phosphorylation Attenuates Hypertrophic Cardiac Growth

The role of GSK3β S9 in cardiac hypertrophy and remodeling has been studied in different in vivo models, including myocardial infarction and TAC. In GSK3α/β-S21A-S9A double knock-in mouse model, where GSK3 is supposed to be constitutively active, preventing hypertrophy, there was no difference in hypertrophic response and remodeling between WT or transgenic mice after permanent MI [36]. Antos et al. showed that in mice with cardiac specific GSK3β S9A knock-in and constitutively activated GSK3β, hypertrophy was diminished in response to chronic beta-adrenergic stimulation with isoproterenol or pressure overload with TAC [37]. In Matsuda et al., GSK3β S9A knock-in model showed reduced cardiac hypertrophy and dysfunction in response to pressure overload induced by TAC [38]. In addition, they utilized GSK3α/β-S21A-S9A double knock-in model, which showed hypertrophic response but preserved left ventricular function after TAC [38].

In cell culture models, hypertrophic agonists such as endothelin-1 increase the phosphorylation of GSK3β Ser9 and thus inhibit GSK3β at least partly, leading to hypertrophic response [39]. In the current study, analysis of GSK3β S389 contribution to hypertrophic cardiomyocyte growth showed that overexpression of GSK3β S389A and GSK3β S9A/S389A significantly augmented basal protein synthesis as well as FGF and insulin-induced hypertrophic response. Overexpression of GSK3β S9A showed some efficacy in attenuating protein synthesis in adult cardiomyocytes, but not in neonatal cardiomyocytes. Moreover, mutation of GSK3β at Ser9 did not rescue the hypertrophic effect of GSK3β S389A, but rather cardiomyocytes overexpressing the double mutant (GSK3β S9A/S389A) showed the most robust hypertrophic response. These data thus indicate that phosphorylation of GSK3β at S389 attenuates cardiomyocyte hypertrophic growth.

Active GSK3 phosphorylates its substrate β-catenin and thus triggers the β-catenin destabilization [40,41]. Inhibition of GSK3 by Wnt leads to stabilized β-catenin, and β-catenin then enters from cytosol to the nucleus and activates gene transcription. This signaling probably does not use the same phosphorylation pathways as Akt mediated signaling. In our studies, cytosolic or nuclear β-catenin levels were not altered by the GSK3β mutations or hypertrophic stimulus.

GSK3β has several targets that play a role in regulation of cardiomyocyte growth. Nuclear GSK3β phosphorylates and inhibits multiple proteins, including transcription factors such as GATA-4, members of the NF-AT family, and c-Myc [42,43,44]. Analysis for nuclear translocation of GSK3β showed that hypertrophic stimulus robustly increases its nuclear translocation. In nonstimulated cardiomyocytes, overexpression of GSK3β S389A slightly reduced nuclear localization of GSK3β when compared to GSK3β S9A, thereby potentially attenuating the inhibitory effect of GSK3β on its nuclear targets. However, mutation of GSK3β at Ser389 did not affect the GSK3β localization in response to hypertrophic stimuli. The enhanced hypertrophic response afforded by GSK3β Ser389Ala thus does not appear to involve regulation of GSK3β nuclear localization. A limitation of our study is the use of overexpression model. Overexpression of proteins may not be sufficient to reveal their biological function as overexpression of a gene results in expression levels exceeding endogenous levels, often by several orders of magnitude. As a result, overexpressed protein may participate in signaling processes in which they are usually not involved.

### 3.3. GSK3β S389 Phosphorylation Attenuates Hypertrophy by Targeting mTORC1 Signaling Pathway

mTOR is a central regulator of cellular energy homeostasis and cell growth [45]. Early studies have shown that inhibition of mTOR with rapamycin attenuates angiotensin II induced activation of p70S6K and angiotensin II induced hypertrophy [46]. In genetic models, manipulation of mTOR complexes has been shown to induce both protective and deleterious effects in the cardiac remodeling, depending on the model [16].

p70S6K is a central downstream effector of mTORC1 [45] and p70S6K has also been shown to phosphorylate GSK3β at Ser9 and to inactivate GSK3β [47]. Interestingly, we found that phosphorylation of p70S6 kinase was increased in cardiomyocytes overexpressing GSK3β S389A or GSK3β S9A/S389A. The role of GSK3β in regulating mTORC1 signaling has also been identified in a previous study showing that GSK3β positively regulates p70S6K activity and cell proliferation [28]. In summary, increased protein synthesis in cardiomyocytes with defective phosphorylation of GSK3β at S389 is associated with activation of p70S6K, thus providing a plausible mechanism for the enhanced hypertrophic growth.

Interestingly, analysis of both human and mouse samples show regulation of GSK3β phosphorylation in the diseased myocardium. Analysis of cardiac samples from patients with end-stage ICM or DCM showed an increase in GSK3β phosphorylation at S9 and S389, and left ventricle samples of mice subjected to TAC showed an increase in GSK3β S389 phosphorylation. Data from our studies with isolated cardiomyocytes suggest that the noted increase in GSK3β S389 phosphorylation may counteract the development of cardiac hypertrophy in the diseased myocardium.

In summary, our data suggest that phosphorylation of GSK3β at serine 389 attenuates hypertrophic cardiomyocyte growth and offers protection from cardiomyocyte death during hypoxic stress. Mechanistically, defective GSK3β S389 phosphorylation is associated with increased phosphorylation of mTORC1 target kinase p70S6K. These data provide novel evidence for the role of GSK3β Ser389 in regulating GSK3β function in cardiomyocytes.

## 4. Materials and Methods

### 4.1. Human Cardiac Samples

The left ventricular samples from patients with ischemic and dilated cardiomyopathy were obtained from patients undergoing cardiac transplantation in Helsinki University Hospital between 2014 and 2019. The investigation conformed to the Declaration of Helsinki, and the protocol was approved by the Ethics Committee of Helsinki and Uusimaa Hospital District, Finland (project identification code 330/13/03/00/13, admitted 12 August 2014). A written informed consent was obtained from the patients. The control samples were collected as part of the FinGesture study, which has systematically collected both clinical and autopsy data from sudden cardiac death victims between 1998 and 2018 in Northern Finland. Control samples were from victims of traffic accidents with no history or evidence of cardiovascular diseases at autopsy. The study complies with the Declaration of Helsinki and was approved by the Ethics Committee of the Northern Ostrobothnia Hospital District, Finland and the National Supervisory Authority for Welfare and Health, Finland (present document number 7204/05.01.00.06/2011, admitted 22 September 2011). Permits to use data from medico-legal death investigations were obtained from the Finnish Institute for Health and Welfare and the Regional State Administrative Agency of Northern Finland.

### 4.2. Hemodynamic Pressure Overload

To induce hemodynamic pressure overload, eight-week-old male C57BL/6 mice were subjected for thoracic aortic constriction (TAC) as described before [48]. The aorta was banded to a size matching a 27G needle. After 6 weeks, heart samples were collected for histology and biochemical analysis.

The experimental protocol was approved by the National Animal Experiment Board (project identification code ESAVI/8134/04.10.07/2017, admitted 6 November 2017). The investigation was carried out in accordance with the national regulations of the usage and welfare of laboratory animals and conforms to the Guide for the Care and Use of Laboratory Animals published by the US National Institutes of Health.

### 4.3. In Vitro Study Design

We used adenoviral vectors for gene delivery and expression of GSK3β as well as its mutants in cultured cardiomyocytes. We used three GSK3β viruses with mutations in phosphorylation sites from serine to alanine: ser9ala (S9A), ser389ala (S389A), and double mutation ser9ala/ser389ala (S9A/S389A). LacZ and wild-type GSK3β were used as control.

### 4.4. Recombinant Adenoviral Vectors

Adenoviruses were generated as described before [49] with modifications. The adenoviruses expressing p38α and p38β have been previously described by Koivisto et al. [50]. To create GSK3β adenoviral vectors, we received pEF5/FRT/V5 mGSK3β 3XFLAG and pEF5/FRT/V5 mGSK3βS389A mutated 3XFLAG plasmids from Brad Doble (McMaster University, Hamilton, ON, Canada), with backbone of plasmid from Invitrogen. Plasmids were transformed in competent XL1Blue cells (Stratagene, San Diego, CA, USA) and plated on ampicillin plates. Minipreps were grown from the bacterial colonies to amplify plasmids. Minipreps were purified with QIAprep Spin Miniprep Kit (Qiagen, Hilden, Germany). S9A mutations were created to both plasmids to obtain four different plasmids: wild-type (WT) GSK3β, GSK3β S9A mutated, GSK3β S389A mutated, and double mutated GSK3β with S9A and S389A mutated. QuickChange II Site-Directed Mutagenesis kit was used (from Agilent Technologies Inc., Santa Clara, CA, USA). Insert was cut out of pEF5/FRTV5 plasmid with KpnI and NotI restriction enzymes (NEB Inc., Ipswich, MA, USA). pShuttle CMV vector plasmid (Qbiogene Inc., Carlsbad, CA, USA) was also cut with same restriction enzymes. Then the cloned insert was ligated to pShuttle CMV plasmid backbone with T4 DNA Ligase (NEB Inc.). Those ligations were again transformed into the XL1Blue cells and they were purified after growing them. Then pShuttle plasmids with GSK3β inserts were linearized with PmeI restriction enzyme (NEB Inc.) and transformed into electroporation competent cells BJ5183-AD-1 (Stratagene, Agilent Technologies). Electroporation was performed using Gene Pulser Transfection Apparatus (Bio-Rad, Hercules, CA, USA) at 2500 V, 200 Ω and 25 μF. BJ5183-AD-1 cells include pAdEasy-1 plasmid that contains most of the human adenovirus serotype 5 genome, but self-replication capacity is prevented. When linearized pShuttle-CMV is transformed in to BJ5183-AD-1 cells including pAdEasy-1 plasmid, homologous recombination is expected to occur, and forms a viral genome including the gene of interest, here GSK3β and its mutated variants. Cells where transformation had occurred were selected by kanamycin resistance and amplified in minipreps. Transformants were purified and homologous recombination was verified with PacI digestion. Then plasmids were transformed into DH5α cells and amplified, and again verified with BstXI digestions. These recombinant adenoviral constructs were then linearized with PacI, purified with ethanol–sodium acetate precipitation, and transformed into AD-293A cells (from Qbiogene Inc.) with Lipofectamine 2000 (Invitrogen, Waltham, MA, USA). These cells produce part of the viral genome E1, which is missing from pAd Easy viral vector, and thus transfecting vector into these cells allows the production of infectious virus particles. A day after transfection, cells began to round up and detach, and all the detached, i.e., infected cells, were collected daily. Cells were centrifuged 1000 rpm for 5 min and resuspended into OptiMEM (Gibco, Waltham, MA, USA). Virus was then released from cells by freeze–thaw cycles and fresh AD-293A cells were infected to multiply virus amount. After four rounds of amplification, viruses were purified and concentrated with 15%:30%:40% ioxidanol density gradient centrifugation (100,000× *g*, at +4 ℃, 24 h). Viruses were diluted in phosphate buffered saline (PBS) and stored at −70 ℃. Titer of viruses was determined by Ad Easy Viral Titer Kit (Stratagene, San Diego, CA, USA). As a control virus, adenovirus containing *E. coli* β-galactosidase coding LacZ gene was used.

### 4.5. Isolation of Neonatal Rat Ventricular Cardiomyocytes

Neonatal rat ventricular cardiomyocytes (NRVMs) were prepared from 2–4 day-old Sprague–Dawley rats, as described earlier [51]. Briefly, rats were sacrificed by decapitation and the thorax was opened to excise the heart. Atria were removed and ventricles were rinsed in PBS and cut in pieces in collagenase type 2 (Worthington Biochemical Corporation, Lakewood, NJ, USA) 2 mg/mL and 25 mM CaCl_2_ in PBS. Cells were fractionated by repeated incubations in collagenase at +37 ℃. After incubations, the cell suspension was centrifuged twice (5 min, 1000 rpm), and the supernatant was discarded and replaced with fresh Dulbecco’s Modified Eagle’s medium/F-12 (DMEM/F-12, Sigma-Aldrich), including 10% fetal bovine serum (FBS, Gibco, Waltham, MA, USA), 2.56 mM L-glutamine (Sigma-Aldrich, Saint Louis, MO, USA), and penicillin–streptomycin 100 IU/mL (Sigma-Aldrich). Isolated cells were preplated for 2 h to remove fibroblasts. Then remnant myocytes were replated at a density of 1.8–2 × 10^5^/cm^2^ and incubated overnight in DMEM/F-12 supplemented with 10% FBS. After that, the medium was changed to complete serum-free culture medium (CSFM; DMEM/F-12, 2.5 mg/mL bovine serum albumin (BSA, Sigma-Aldrich), 1 μM insulin + 32 nM selenium + 2.8 mM sodium pyruvate + 5.64 μg/mL transferrin (insulin-transferrin sodium-selenite media supplement, Sigma-Aldrich), 1 nM 3′-3′-5′triiodotyronine (Sigma-Aldrich), 2.56 mM l-glutamine, 100 IU/mL penicillin–streptomycin). After experiments, medium samples were collected and wells were rinsed twice with cold PBS and quickly frozen at −70 °C.

### 4.6. Isolation of Adult Rat Ventricular Cardiomyocytes

Adult rat ventricular cardiomyocytes (ARVMs) were isolated from 8–12 week-old male Sprague–Dawley-rats as described before [51] and modified from [52]. Briefly, the rat was euthanized with CO_2_ and the heart was excised rapidly. The heart was cannulated through aorta and retrogradily perfused with Hepes-buffered Tyrode’s solution supplemented with 1 mg/mL collagenase type II (Worthington Lakewood, NJ, USA) and 2,3 -butandione-monoxime (BDM, Sigma-Aldrich) until digested. Ventricular tissue was homogenized, and myocytes were collected with low-speed centrifugation. After Ca^2+^ reintroduction, cardiomyocytes were resuspended and cultivated in αMEM supplemented with Earle’s salt (Invitrogen, Waltham, MA, USA) containing 5% FBS (Gibco, Waltham, MA, USA), 20 mM HEPES (Sigma), insulin-transferrin-selenium (Gibco), 10 mM BDM, 2 mM l-glutamine and penicillin–streptomycin in laminin-coated (10 μg/mL, Sigma-Aldrich) plates at a density of 1–1.5 × 10^4^/cm^2^. After the cells had attached, medium was changed, and FBS was replaced with 0.01% BSA when starting the experiment. After experiments, medium samples were collected, and wells were rinsed twice with PBS and quickly frozen at −70 °C.

### 4.7. Adenoviral Infections

Penicillin-streptomycin was left out of the medium where viruses were added to cells. In NRVM cultures, adenoviruses were added at 3 MOI 24(-48) h after plating, depending on the designed experiment. In ARVM cultures, adenoviruses were added at 100 MOI 2 h after plating.

### 4.8. Measurement of Protein Synthesis

Cardiomyocyte protein synthesis was measured by analyzing a radioactively labeled [^3^H]-leucine incorporation into the cells. NRVM or ARVM were cultured in 24-well plates, and when hypertrophic stimulus was added, medium was supplemented with [^3^H]-leucine (5 µCi/mL). After 24 h cells were lysed and processed for measurement of incorporated [^3^H]-leucine (AmershamPharmacia Biotech, Piscataway, NJ, USA) by liquid scintillation counter (PerkinElmer Tri-Carb 2900TR, Liquid Scintillation Analyzer with Quanta Smart™ 2.03 Software).

### 4.9. Hypoxia–Reoxygenation in Cell Culture

ARVM cells were infected with viruses 2 h after plating. After 24 h, medium was changed to plainer version without FBS, penicillin–streptomycin, and HEPES. The cells were incubated in hypoxic C-Chamber with oxygen levels controlled at 0.1% in 5% CO_2_ with ProOx C21 O_2_/CO_2_ controller (BioSpherix, Parish, NY, USA) for 4 h and after were allowed to reoxygenate for 3 h. Medium samples for cell viability assay were collected after reoxygenation.

### 4.10. Cell Viability Assay

Analysis of necrotic cell death was performed by measuring the release of adenylate kinase from ruptured ARVM into the cell culture medium by using a bioluminescent ToxiLight bioassay (Lonza, Basel, Switzerland) according to manufacturer’s instructions.

### 4.11. Western Blot Analysis

Frozen cells, scraped from cell culture plates and frozen cardiac tissue samples that were ground in liquid nitrogen, were dissolved and homogenized in ice-cold lysis buffer containing 20 mM Tris-HCl, 150 mM NaCl, 1 mM EDTA, 1 mM EGTA, 1% (*v*/*v*) Triton-X100, 2.5 mM sodium pyrophosphate, 1 mM β -glycerophosphate, and 1 mM Na_3_VO_4_ (pH 7.5) supplemented with 1 mM dithiothreitol (DTT, 1:1000), protease inhibitors (1:100), and phosphatase inhibitors (1:100, Sigma-Aldrich). Samples were then centrifuged at 10,000× *g* for 5 min at 4 °C and the supernatant was collected. Protein concentrations were determined by the Bradford method. Protein extracts were matched for protein concentration and stored denatured in SDS loading buffer at −70 °C. Nuclear and cytosolic proteins were extracted as described [53]. Equal volumes (20–50 µg) of protein samples were loaded onto 12–14% SDS-PAGE and transferred to nitrocellulose membranes. Antibodies and their dilutions used were phospho-GSK3β (Ser389) (07-2275, Millipore, 1:1000), phospho-GSK-3beta (Ser389) (14850-1-AP, Proteintech, 1:1000) phospho-GSK-3β (Ser9) (#9336, Cell Signaling, 1:1000), GSK3β (27C10) (#9315, Cell Signaling, 1:1000), phospho-Akt (Ser473) (#9271, Cell Signaling, 1:1000), phospho-p70 S6 Kinase (Thr389) (#9205, Cell Signaling, 1:1000) β-Catenin (610154, BD, 1:1000), and Vinculin (ab18058, Abcam, 1:1000), Lamin B (C-20) (sc-6216, Santa Cruz, 1:1000) or Glyceraldehyde-3-Phosphate Dehydrogenase (MAB374, Merck, 1:100000) was used as a loading control. Secondary antibodies were purchased from Life Technologies (Alexa Fluor A11371, A21058, and A21076) and used in the dilutions of 1:5000. Antibodies were diluted in Odyssey Blocking Buffer from LI-COR. Protein levels were detected using fluorescence with Odyssey Fc imaging system (LI-COR Biosciences, Lincoln, NE, USA).

In Western blots for GSK3β bands, two bands are detected when cells are infected with GSK3β viruses; lower band is endogenous GSK3β and upper band is viral GSK3β, since it is slightly heavier including 3xFLAG Tag.

### 4.12. Mitochondrial Membrane Potential Assay

For the analysis of mitochondrial membrane potential, ARVM were incubated with 1 µM JC-1 dye from Millipore (Merck KGaA) for 30 min at +37 °C. Cells were washed once with cell culture medium and hypoxia experiment was performed as described above. After hypoxia, fluorescent readings for JC-1 aggregate emission (590 nm) and JC-1 monomer emission (530 nm) were measured with Varioskan Flash (Thermo Scientific) using Skanlt Software for Varioskan Flash version 2.4.5 (Thermo Scientific).

### 4.13. Statistical Analysis

Statistical analysis was performed with IBM SPSS Statistics software. To compare multiple groups, one-way ANOVA was used, followed by Tukey post hoc test for equal variances, or the Games–Howell post hoc test for unequal variances. The Kruskall–Wallis test was performed when data did not represent normal distribution. Normality of variables was tested with Kolmogorov–Smirnov and Shapiro–Wilk tests. When two groups were compared, Student’s *t*-test or Mann–Whitney U-test was performed. Data are shown as mean ± SD. Differences were considered statistically significant at the level of *p* < 0.05.

## Data Availability

Not applicable.
